# Dissecting role of founder mutation p.V727M in GNE in Indian HIBM cohort

**DOI:** 10.1515/med-2021-0391

**Published:** 2021-11-15

**Authors:** Shivangi Attri, Vikas Sharma, Amit Kumar, Chaitenya Verma, Suresh Kumar Gahlawat

**Affiliations:** Department of Biotechnology, Chaudhary Devi Lal University, Sirsa, 440002, India; General Facility, Centralized Core Research Facility, All India Institute of Medical Sciences, New Delhi, 110029, India; ICMR-AIIMS Computational Genomics Centre, Division of Biomedical Informatics, Indian Council of Medical Research, New Delhi, 110029, India; Department of Pathology, Wexner Medical Center, The Ohio State University, OH-43210, Ohio, United States of America; Department of Biotechnology, Chaudhary Devi Lal University, Sirsa, Haryana, 125055, India

**Keywords:** HIBM, GNE, myopathy, simulation, founder mutations

## Abstract

GNE gene-specific c.2179G>A(p.V727M) is a key alteration reported in patients with hereditary inclusion body myopathy (HIBM) and represents an ethnic founder mutation in the Indian cohort. However, the underlying role of this mutation in pathogenesis remains largely unknown. Thus, in this study, we aimed to access possible mechanisms of V727M mutation that could be leading to myopathy. We evaluated various *in silico* tools to predict the effect of this mutation on pathogenicity, structural or possible interactions, that could induce myopathy. Our results propose that V727M mutation could induce deleterious effects or pathogenicity and affect the stability of GNE protein. Analysis of differential genes reported in the V727 mutant case suggests that it can affect GNE protein interaction with Myc-proto-oncogene (MYC) transcription factor. Our *in silico* analysis also suggests a possible interaction between GNE ManNac-kinase domain with MYC protein at the C-terminal DNA-binding domain. MYC targets reported in skeletal muscles via ChIP-seq suggest that it plays a key role in regulating the expression of many genes reported differentially expressed in V727M-mutated HIBMs. We conclude that V727M mutation could alter the interaction of GNE with MYC thereby altering transcription of sialyltransferase and neuromuscular genes, thus understanding these effects could pave the way for developing effective therapies against HIBM.

## 1 Introduction

Hereditary inclusion body myopathy (HIBM), also known as Nonaka myopathy or GNE myopathy, is rare autosomal recessive disease caused by defects in muscles leading to progressive skeletal muscle atrophy. It was initially reported by Nonaka et al. in Japan and Argov and Yarom in Israel [1–3]. HIBM starts in the stage of young adulthood, with the most common symptoms arising in the second and third decades. The first sign of this myopathy may include features like weakness in distal limb muscles. This disease involves a bilateral foot drop caused by weakness of the anterior tibialis muscles with onset in early adulthood. It slowly progresses to involve skeletal muscles throughout the body over the next decades and leads to relative sparing of the quadriceps until the late stages of the disease. Post-diagnosis, such patients keep on attaining severe disability up to 10–20 years, which leads them to be confined with wheelchairs and other support systems [4].

The prevalence of HIBM is estimated at ∼ 1 to 9/1,000,000 (Orphanet; http://www.orpha.net/). By 2001, mutation in the UDP-*N*-acetyl-glucosamine-2-epimerase/*N*-acetylmannosamine kinase gene (GNE) was identified as the key cause of HIBM, thereby confirming this to be a monogenic disorder [5]. As per ensembl database, six different GNE transcripts are reported for human (Figure S1). Such GNE transcripts encode a bifunctional enzyme known to encode a rate-limiting step which plays a key role in sialic acid synthesis [6]. Mutation in the gene is known to lower sialic acid levels, especially on the surface of certain proteins involved in muscle functioning. Thus, patients with HIBM are unable to produce sufficient amount of sialic acid for normal cellular function leading to muscle-wastage-induced disability.

Although hyposylaiton of such muscle glycans is considered as a major cause of this disorder, molecular and pathophysiological cause of the disease is still not clear. Further, multiple GNE mutations have been reported across various countries [4,7,8]. Interestingly, a large set of these mutations have been reported in the ManNAC kinase-specific C-terminal region, which seems to be an important structural region (Figure S2). Further, many of these mutations have been found to be abundantly prevalent specific to the population of certain regions [8]. Interestingly, p.V727M mutation of hGNE2 (also represented as V696M in hGNE1 transcript) is one such important mutations that is reported in different parts of the world, including Thailand, India, and Algeria [5,9,10,11]. Various studies show that this mutation is considered as a European Roma Gypsies-specific mutation [12,13]. This mutation is highly prevalent in Indian population, thus the leading theory suggests that this mutation was an ethnic founder mutation originated in India that slowly spread to the Eastern Europe during the 13–14th centruries AD. Although the V727M mutational role in inducing HIBM is known for long, the exact role of this mutation in inducing myopathy still remains elusive. This mutation has become of high clinical significance as it is present in greater than 65% of the Indian HIBM cohort [13]. As nearly all mutations reported in GNE tend to induce defective protein rather than any decay of GNE mRNA, thus V727M-associated mutation probably seems to act by altering structural and/or molecular functions via an unknown mechanism [14]. Thus, in this study, we explored the *in silico* approach to identify V727M-induced functional and phenotypic changes using MutPred and PredictSNP. To explore further the vibrational entropy and enthalpic changes of flexible conformations, DynaMut was used, which is a consensus predictor that integrates normal mode approaches with graph-based distance matrix in the mutating residue environment. Simulation studies were also performed to study the effect of V727M mutation on the structure of the protein. We also explored tools to study protein–protein interaction and available ChIP-seq data to analyze possible interactions. Findings of this study suggests a possible interaction between ManNac kinase domain of GNE with the Myc-proto-oncogene (MYC) protein. We suggest that HIBM-specific V727M mutation may impair GNE protein interaction with MYC protein. Such interactions could be altering transcription of sialyltransferase (STs) and neuromuscular-disease-associated genes leading to HIBM myopathy.

## 2 Materials and methods

### 2.1 Sequence download using UniProt and multiple sequence alignment

GNE gene and protein sequences were obtained from various organisms using NCBI Gene Bank and UniProt, respectively. Sequences were next aligned using Clustal Omega program. GNE amino acid-specific sequences of the C terminal region (representing 671–722 aa of hGNE1 or 702–753 aa of hGNE2) were aligned to analyze the sequence identity and divergence in this region across various species. As per recommended international nomenclature standards, the key mutation under study – dbSNP: rs121908627 (representing V696M mutation of hGNE1, or V727M as per hGNE2 transcript) will be preferably represented as V727M in rest part of the study.

### 2.2 Mutation effect prediction on function and phenotype

The effect of GNE gene-specific V727M mutation on phenotype and functions was analyzed using multiple platforms, including MutPred and PredictSNP tools. To identify the impact of non-synonymous single nucleotide polymorphisms (SNPs) on protein structure stability, I-Mutant and MUpro prediction tools were used on protein sequence at pH 7.0 and temperature 25°C [15,16].

### 2.3 PROVEAN analysis

PROVEAN is an important tool that predicts the amino acid substitution effect on the biological function of the protein of interest. PROVEAN tool is used to perform BLAST hits clustering, and to generate the final PROVEAN score, a delta alignment score is calculated for each supporting sequence and then averaged in and across clusters [17]. As per PROVEAN analysis, a default score of −2.5 or higher is considered deleterious, whereas all other scores are neutral.

### 2.4 SNAP2 tool analysis

The functional impact of single amino acid substitution in the GNE gene at V727M was evaluated by using a neural network-based tool known as SNAP2 (https://rostlab.org/services/snap2web/). The SNAP2 server predicts whether any SNP is likely to alter protein function by analyzing various SNPs biophysical characteristics, structural properties, and evolutionary information. The generated results include a score (ranging from −100 to 100) and a prediction (effect or neutral). Score value between −100 and 0 is considered to indicate a neutral prediction, while 1–100 indicates an effect [18].

### 2.5 NetSurfP analysis

The web-based NetSurfP-2.0 server (http://www.cbs.dtu.dk/services/NetSurfP) was used in this study to predict protein solvent accessibility and the secondary structure. NetSurfP can also predict reliability as a *Z*-score for each of the predictions, in addition to the absolute solvent accessibility (ASA) and secondary structure prediction [19]. Here the normal and predicted SNP sequences in FASTA format were submitted for prediction to the NetSurfP.

### 2.6 Changes in vibrational entropy and normal mode analysis

The effects of the V727M (V696M as per hGNE1) mutations in flexible conformations on GNE protein stability (PDB ID 2YHY) were analyzed using DynaMut tool, which is a consensus predictor of protein stability and based on the vibrational entropy changes predicted using ENCoM and stability changes predicted by the graph-based signature approach of mCSM. The performance of DynaMut tool outperforms alternative algorithms that also provide measurements of the effects of single-point mutation on protein stability.

### 2.7 Molecular dynamics simulation of wild type (WT) and mutant structure

The structure of the WT *N*-acetylmannosamine kinase was obtained using Protein Data Bank (PDB ID: 2YHW, https://www.rcsb.org/). One monomer with the bound Zn ion was extracted from it and was used as the starting structure for MD simulation. The mutant structure was prepared by introducing the V727M (V696 as per hGNE1) mutation in the WT structure using the rotamer library in UCSF chimera [20]. The protein and the parameters of the ion were taken using ff14SB force field [21]. The system was kept at the center of a cubical box with a padding distance of 15 angstrom and solvated using TIP3P water model. After solvation, the appropriate number of Na^+^ and Cl^−^ ions was added to the system by replacing the water molecules to neutralize the system and maintain the ion concentration of 0.15 M. Subsequently, energy minimization and two phases of equilibration (first constant number, volume and temperature [NVT] at 300 K temperature followed by constant number, pressure and temperature [NPT] at 300 K temperature and 1 bar pressure) were performed. Finally, the production run in NPT was run for 50 ns. The system preparation and MD simulations were all done using GROMACS version 2019.2 (http://doi.org/10.5281/zenodo.2636382). To analyze the simulation results, root mean square deviation (RMSD) and root mean square fluctuation (RMSF) were analyzed for simulation time (50 ns), which represent RMSF (in Angstrom) vs residues.

### 2.8 Post-translational modification analysis at target genes

To identify whether GNE V727M mutation induces any post-translational modification at the GNE protein, we analyzed potential post-translational modification sites at GNE using iPTMnet, MetOSite, and PhosphoSitePlus databases. All these databases were analyzed for key modifications, including phosphorylation, acetylation, sumoylation, and ubiquitinations. Further, the location of these modifications was also accessed with respect to V727M mutations at the GNE gene.

### 2.9 GNE V727M-induced differential genes and pathway analysis

The Pubmed and gene expression omnibus database were searched to find studies reporting expression profiling data on V727M mutant human patients or cell lines. The differentially expressed genes so identified were next analyzed using GeneCodis tools [22]. GeneCodis is a web-based tool that effectively integrates different sources of information and provides search for annotations that frequently co-occur in a set of genes and rank them by their statistical significance. The analysis of concurrent annotations provides significant information for the biological interpretation of different parameters. GeneCodis tool was used to analyze the key pathways affected as well as to identify key transcription factors (TFs) regulating these genes.

### 2.10 GNE transcription factor analysis

To identify the key TFs that were differentially expressed in V727M mutant cells, the BioGRID tool was accessed for GNE protein interacting partners. This is an online interaction repository with data compiled through comprehensive curation efforts. We also analyzed reported human genes encoding TFs and chromatin-modifying proteins that have been identified and used by Ignatieva et al. [23]. We used a tool known as Predict associated TFs from annotated affinities (PASTAA) to predict various TF-regulating differentially expressed genes reported in V727M mutant cases as reported by Chakravorty et al. [22].

### 2.11 Molecular docking study using ClusPro

To study the stable protein–protein interaction and related sub-cellular functions, molecular docking analysis was performed. For this, the ClusPro (version 2.0): protein–protein docking server was used. In ClusPro server, the PIPER docking program is used in the rigid body docking phase, which relies on the fast Fourier transform correlation approach. PIPER represents the interaction energy between two proteins using an expression of form *E*; *E* = *w*_1_ *E*_rep_ *+ w*_2_ *E*_attr_ *+ w*_3_ *E*_elec_ *+ w*_4_ *EDARS,* where *E*_rep_ and *E*_attr_ denote the repulsive and attractive contributions to the Vander Waals interaction energy, and *E*_elec_ represents the electrostatic energy. *DARS* represents a pair-wise structure-based potential. It primarily represents desolvation contributions, which is the free-energy change observed by the removal of water molecules from the interface. The coefficients *w*_1_, *w*_2_, *w*_3_, and *w*_4_ in this method define the weight of the corresponding residues.

### 2.12 MYC protein target analysis in skeletal muscle cells

For the understanding role of MYC in the regulation of gene expression, the reported genome-wide binding profile of c-MYC in skeletal muscle cells was analyzed. For this, a study by Luo et al. provided global insight of the role of c-MYC in myoblast differentiation via ChIP-seq studies in both myoblasts and myotube cells [24]. The differential genes reported in patients with V727M-mutated HIBM were next evaluated to analyze whether their regulation was by MYC transcription factor.

## 3 Results

### 3.1 GNE V727M-specific region is conserved across species

Gene ID for GNE gene is 10020 in the NCBI database, whereas its protein accession is NP_001121699 (for 753 aa protein) and the UniProt KB ID for this protein is Q9Y223. In ensemble human database, six different *GNE* transcripts have been reported, out of which five are the protein-coding ones (Figure S1). This includes originally identified GNE transcript (Ensembl: ENST00000377902) that encodes 722 aa protein as well as the longest transcript (Ensembl: ENST00000396594) encoding 753 aa protein. Interestingly, these two transcripts are the most studied GNE forms that share the 12 (out of 13) exons, but vary only in the alternative first exonic region. Due to this, the overall 753 aa GNE transcript (Ensembl: ENST00000396594) is 31 amino acids longer than the originally reported 722 aa transcript (Ensembl: ENST00000377902). Hence, the V696M mutational region of interest in original transctipt1 is 31 nt far and designated as V727M in longer transcript. To avoid the confusion in mutation nomenclature, we adopt V727M (dbSNP: rs121908627) as the Indian ethnic founder mutation in this study. Multiple sequence alignment of protein sequence corresponding to all transcripts and UniProt KB entry was performed using Clustal Omega. Our analysis showed that 696th position (dbSNP: rs121908627) of transcript hGNE1 (corresponding to V727M of hGNE2 transcript) is conserved across species. Our sequence analysis in a set of 15 organisms using Clustal Omega reported GNE region across V696 to be highly conserved across a large set of species (Figure S3a). Phylogenetic tree analysis showed GNE region to be more close to Aotus and Rhinopithecus as compared to Felis and alligators, although, overall, the region seems to be conserved (Figure S3a and b).

### 3.2 V727M in GNE may induce a pathogenic effect

**Figure 1:**
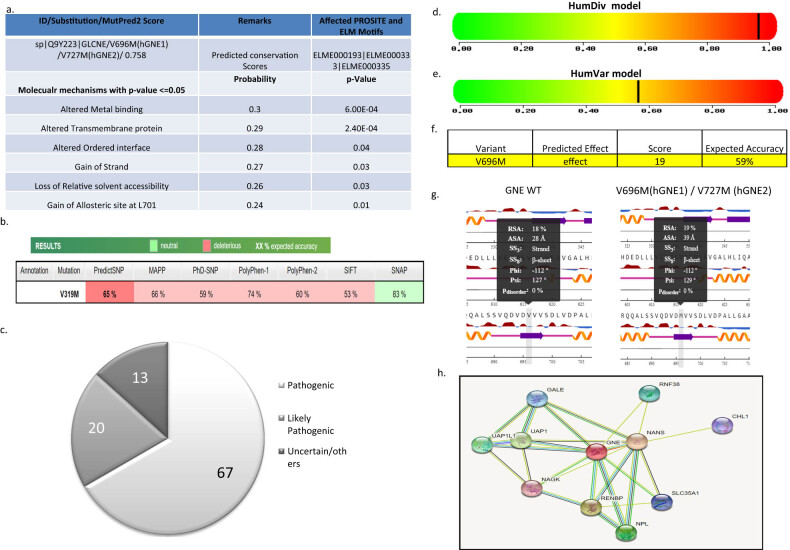
GNE V727M mutation effect prediction on function and phenotype-associated properties using (a) MutPred; (b) PredictSNP; (c) ClinVar-based study; (d) HumDivmodel; (e) HumVar model; (f) SNAP2 analysis; (g) NetSurfP analysis; and (h) susceptible interacting partners (as per STRING analysis).

To understand the role of GNE-specific V727M (V696M in hGNE1) mutation on altering protein function and phenotype, MutPred and PredictSNP-based analysis approaches were used to define the severity pathogenesis (Figure 1a–b). MutPred [25] server was used to predict the pathogenicity upon mutation. Our analysis using MutPred showed that the mutant V319M (representing V727M) could predicatively induce changes that could be inducing altered metal-binding (Pr = 0.30 | *P* = 6.0 × 10^−4^) and altered transmembrane protein (Pr = 0.29 | *P* = 2.4 × 10^−4^) (Figure 1a). Other changes reported less significant effect included loss of relative solvent accessibility (Pr = 0.26 | *P* = 0.03) and gain of allosteric site at L701 (Pr = 0.24 | *P* = 0.01). We also used PredictSNP tool, which is a consensus classifier combining eight prediction methods, namely MAPP, nsSNPAnalyzer, PANTHER, PhD-SNP, PolyPhen-1, PolyPhen-2, SIFT, and SNAP [26]. Our analysis using PredictSNP also showed that the induced mutation had an overall 65% deleterious effect, thus affecting the overall stability of the protein (Figure 1b). Further, to confirm the above results, we searched for V727M-specific mutations reported in ClinVar database (https://www.ncbi.nlm.nih.gov/clinvar/variation/6028/). Based on this approach, we identified 15 submissions for GNE for p.Val696Met mutation (till the year 2020). Interestingly, 13 of 15 of these studies reported this type of mutation to be either pathogenic or likely pathogenic in nature (Figure 1c). Overall the MutPred, PredictSNP, and ClinVar data analysis suggests V727M mutation could be inducing alterations in the GNE functions.

### 3.3 GNE mutation may affect protein stability

I-Mutant analysis also showed that V727M mutation under mentioned conditions decreases stability (DDG value prediction: −1.14 kcal/mol) (Figure 1d). Similarly, MuPro predicted that mutation might be leading to decreased stability (∆∆G = −0.90102119) (Figure 1e). Analysis of PROVEAN tool showed that V727M amino acid substitution is non-deleterious (score −0.52, prediction-neutral, data not shown), whereas the SNAP2 tool analysis showed a score of 19, thereby predicted this mutation to alter protein functions (Figure 1f). NetSurfP tool was also used to predict the accessibility of solvent and secondary structure of the protein. Our analysis showed that V727M mutation induced no change in the protein secondary structure; however, an increase in ASA was observed due to this mutation (from 28 to 39 A°; Figure 1g). The effect of stability was important as GNE could likely have many interacting partners (as per STRING tool analysis) whose downstream signaling could be affected due to decreased stability (Figure 1h). Interestingly some of these interactors, for example, UAP1, RNF38, and NANS protein, were also localized in the nucleus suggesting GNE could be playing an essential role in effecting nuclear signaling.

### 3.4 V727M mutation may affect vibrational energy

**Figure 2:**
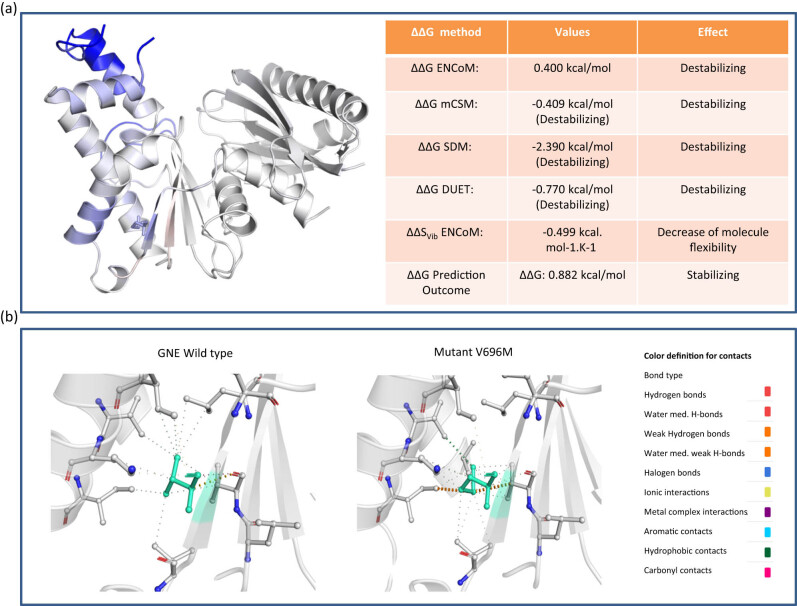
DynaMut analysis results: (a) ΔG prediction outcome using different methods (amino acids colored according to vibrational entropy change upon mutation, here blue color represents a rigidification of the structure); and (b) interaction associated changes in GNE WT and Mut forms.

For exploring vibrational entropy and normal mode analysis, DynaMut prediction method was used. This method integrates the output from ENCoM and several other predictors into its score for identifying the vibrational energy changes. The performance of DynaMut tool was used as it outperforms alternative algorithms that also provide measurements of the effects of single-point mutations on protein stability. The finding of this study was analyzed to study the impact of this mutation on parameters including vibrational energy, interatomic interaction effect, atomic fluctuation, deformation energy study, and fluctuation analysis (Figure 2a–b, Figure S4a–c). Our analysis showed that V727M mutation in GNE gene induces most of these predictors to show destabilizing alterations in vibrational energy (Figure 2a). Further, our assessment showed that Val696 (left) to Met(right) could also affect protein stability due to changes in interatomic interactions (Figure 2b).

### 3.5 Simulation studies show no effective change by V727M mutation in GNE

**Figure 3:**
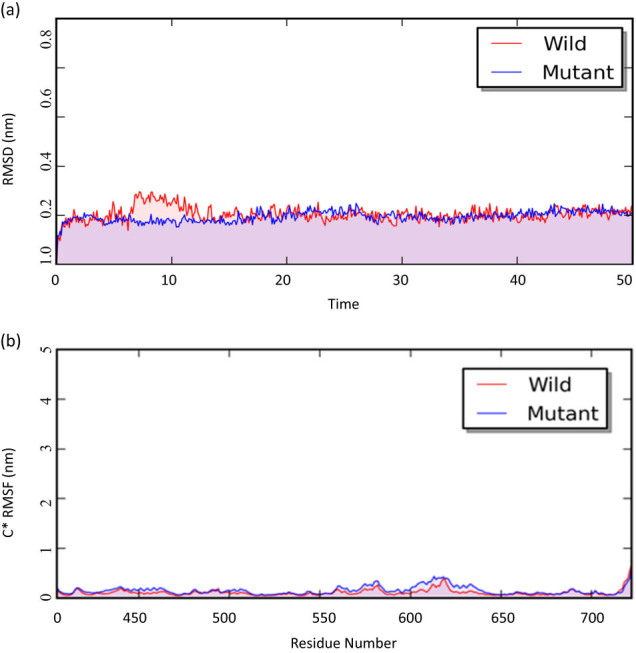
Simulation results: (a) RMSD at 50 ns time interval; and (b) RMSF results for the 400–722 aa residue of the GNE transcript hGNE1.

Analysis of single nucleotide mutation effect (V727M) on the structure and function, molecular dynamics simulation was performed. For this, the PDB ID 2YHY protein structure was considered WT and the mutant protein structure was developed using the UCSF Chimera visualization tool. Further, the interaction of this protein and its effect in inducing disease phenotype were also studied. Molecular dynamics simulation was performed to analyze the effect of single nucleotide mutation (V727M) on the structure, function, and interaction of this protein. Production was carried out for 50 ns. For 50 ns-based analysis, a time step of 2fs was considered. Overall, 5,000 frames were generated (saving frequency of 10 ps). To analyze the simulation results, RMSD was calculated for simulation time (50 ns) (Figure 3a and b). RMSD plot represents RMSD (in Angstrom) versus simulation time (in ns). Our analysis using RMSD plots for WT and mutant (V319M) *N*-Acetylmannosamine kinase (PDB: 2YHY) showed that no significant structural variation was present in mutant protein as compared to the WT protein for overall simulation time (50 ns) (Figure 3a). Thus, the possible effect due to V727M mutation at GNE gene could not interpret it to be inducing a destabilization effect on the structure of GNE protein. We also analyzed RMSF for simulation time (50 ns), which represent RMSF (in Angstrom) vs residues. Our study based on RMSF plots for WT and mutant (V319M) *N*-acetylmannosamine kinase (PDB: 2YHY) also showed no effective fluctuation was observed in mutant protein structure as compared to the WT protein for most of the residues (Figure 3b).

### 3.6 GNE V727M do not induce post-translational modification effects

To identify whether GNE V696 (or V727M region of hGNE2) mutation induces any post-translational modification at the GNE protein, we analyzed the key post-translational modification sites at GNE. All the three databases (iPTMnet, MetOSite, and PhosphoSitePlus) used in this study showed the presence of various phosphorylation, acetylation, and ubiquitination modifications at the GNE protein. However, none of the modifications were present in the 15 aa range near the V727M mutation site (Figure S5). Thus, we concluded that V727M mutation does not seem to affect GNE protein via inducing any post-translational modifications.

### 3.7 GNE V727M affect neuromuscular and metabolism-associated genes in HIBM disorder

**Figure 4:**
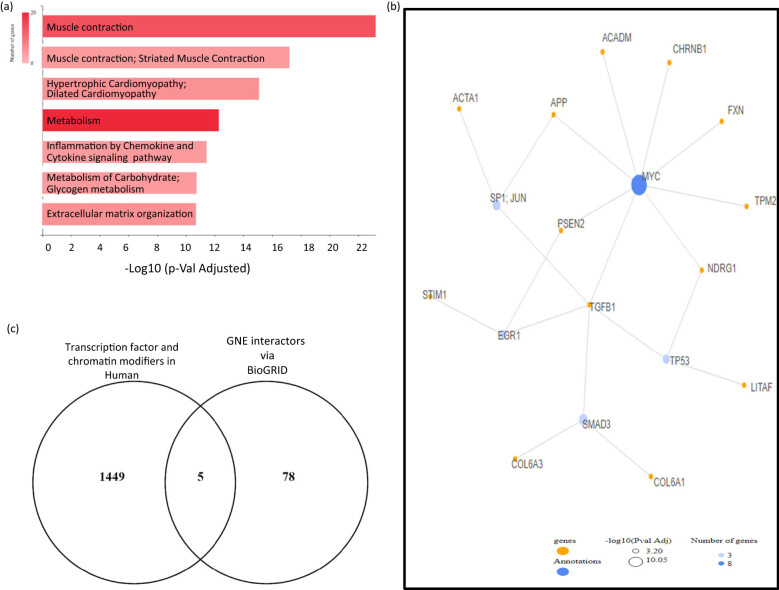
(a) The reported differential genes in patients with V727M mutant were analyzed using GeneCodis to identify key pathways involved. (b) Key transcription pathways and the tartget genes regulated as per GeneCodis tool. (c) Common TFs identified via human transcription factor or chromatin modifiers and BioGRID-based GNE protein interaction network.

Since V727M mutation does not seem to affect the structure or post-translational modification of the GNE protein, we hypothesized the importance of affecting any protein–protein interaction. To identify the key genes affected due to V727M mutation, a literature search was performed. Interestingly, a recent study by Chakravorty reported altered genes in V727M mutant patients using RNA-sequencing approach. The reported differential genes were analyzed using GeneCodis to identify differential pathways. Our analysis of these genes showed that top-altered seven pathways found affected associated with muscle contraction, carbohydrate and glycogen metabolism, and extracellular matrix organization (Figure 4a). We also identified key TFs that could potentially be regulating these targets using GeneCodis. Interestingly MYC, SMAD3, JUN, EGR1, and TP53 were found to be the key TFs that seem to be regulating this set of genes (Figure 4b). Interestingly, MYC was found to be the most prominent transcription factor among these.

### 3.8 GNE interacting protein expression could be regulated by MYC

To analyze the key known human genes encoding TFs and chromatin-modifying proteins affected by V727M mutation in GNE gene, the reported list of modifiers was obtained using data by Ignatieva et al. [23]. Next, the BioGrid tool was used to identify susceptible protein–protein interactors with GNE. The common targets were checked to identify the potential TFs. Our analysis led to the identification of five common elements (HOXA1, MYC, ZNF213, ZNF764, and GABPA) (Figure 4c and Figure S6). Interestingly, out of these, MYC was the only transcription factor target that was found to be common with those regulating differentially expressed genes in V727M patient-based RNA-seq-based data (Figure 4b). To confirm the same, we also used PASTAA program to predict TF-regulating differential genes reported in V727M-mutated cases. Our PASTAA-based analysis also suggests MYC to be one of the key regulators of the genes sets that were differentially expressed in V727M-mutated case (data not shown).

### 3.9 GNE may interacts with MYC protein via its ManNAc domain

**Figure 5:**
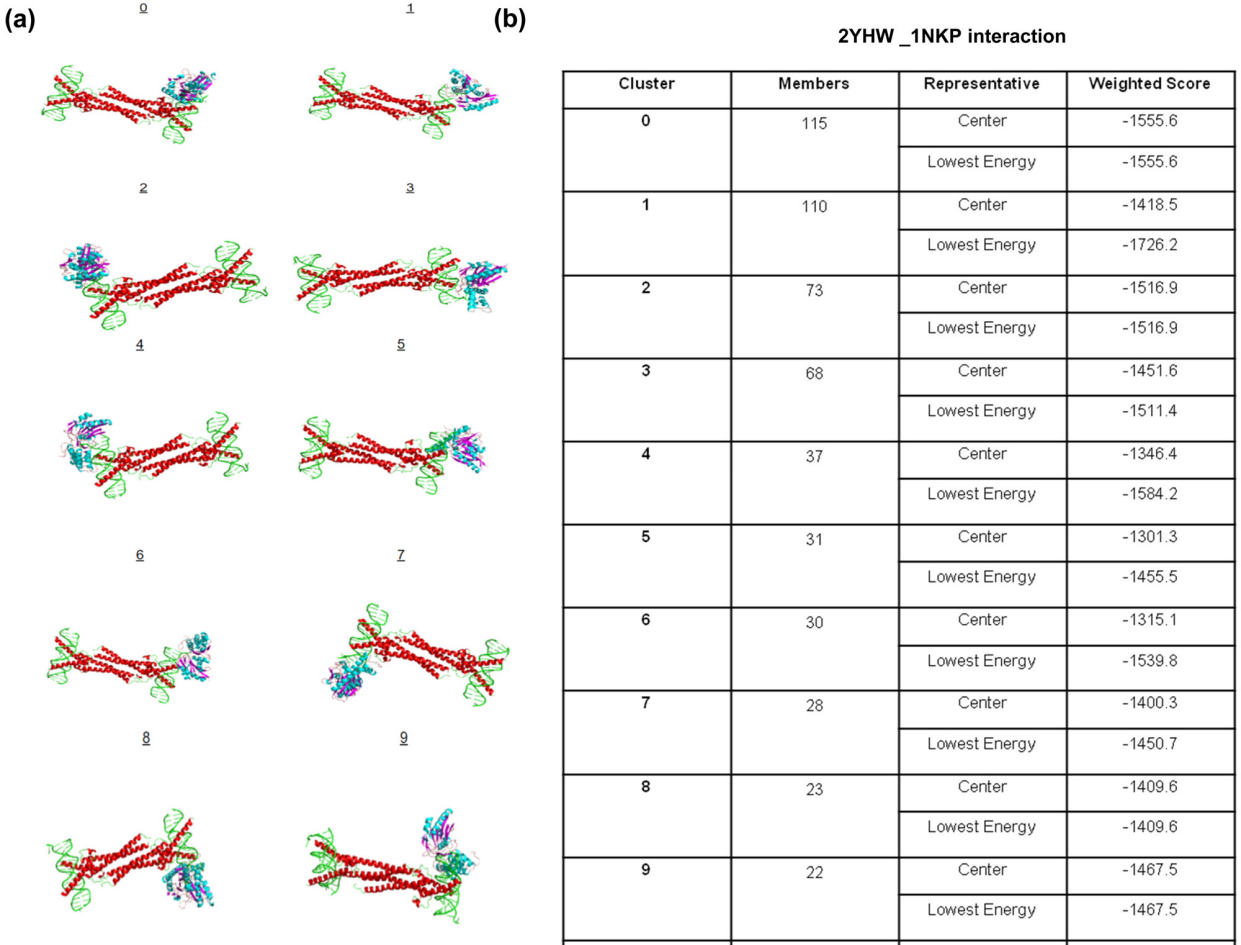
ClusPro-based analysis of molecular docking results: (a) top 10 predicted interactions of GNE protein domain (2YHW) and the MYC domain (PDB entery 1NKP) and (b) cluster scores for each predicted interaction.

Our previous results show MYC could be an important transcription factor that may also be an active interactor of GNE protein. To confirm this, we used ClusPro 2.0 tool, which compares and validates the accuracy, stable protein–protein binding, and molecular interactions. Thus, to validate an interaction between the ManNac kinase domain and the MYC protein-specific domains, the 2YHW PDB structure of GNE domain was analyzed for its interaction with all known reported PDB structures for MYC. Interestingly, molecular docking was significant with a high negative energy value (−1555.6 kcal/mol) of the top ordered protein–protein docking complex between GNE protein domain (2YHW) and the MYC domain (PDB entry 1NKP, A/D chain, 353–434 position), suggesting a high probability of protein–protein interaction (Figure 5a and b). Similarly, a significant high negative energy (−1577.0 kcal/mol) for the docking between GNE protein domain (2YHW) and the MYC domain (PDB entry 5I50, A/B chain, 350–439 position) also suggested such interaction (Figure S7a and b). Interestingly, this region at MYC protein is found to have multiple post-translation modifications, including sites for ubiquitination, phosphorylation, acetylation, and sumoylation. We hypothesize that the GNE protein-specific V727M mutation could be affecting the overall interaction with MYC gene at these regions and could also be affecting post-translational effects.

### 3.10 MYC may bind chromatin and regulate HIBM-associated genes in skeletal muscles

To understand the role of MYC transcription factor in skeletal muscles, we searched published studies that used high-throughput ChIP-seq-based approach to identify c-MYC target, especially in the muscle cells associated with HIBM disorders. In one such important study by Luo et al., a global insight of the role of c-MYC was analyzed by ChIP-seq approach in myoblast as well as myotube cells [24]. We analyzed the genes in MYC antibody precipitated ChIP-seq data and the differential genes reported by Chakravorty et al. (2019) in patients with V727M-mutated HIBM [24,27]. Zuo et al.’s analysis shows a large number of MYC targets were reported via ChIP-seq in myoblasts as compared to myotubes, thus indicating an active role of MYC in gene regulation in myoblast cells. Interestingly, about 37% (33 of 89) of differential genes in Chakravorty et al.’s study had MYC occupancy in myoblasts targets, whereas only 10% of such genes (9 of 89) had MYC occupancy in myotube cells (Table S1). Interestingly, so identified 33 and 9 genes were completely different in myoblast and myotube cells, thereby suggesting that MYC could regulate relatively different biological processes in myoblast cells compared to myotubes. This also suggests that possibly different pathways are regulated by MYC in different tissues in HIBM myopathy, which needs further investigation.

## 4 Discussion

GNE-associated V727M mutation has been considered as an important pathogenic mutation causing HIBM myopathy in India. Its unusual high frequency in the Indian cohort as compared to other parts of the world suggests that it is more likely to be a founder mutation. Further, few studies have previously suggested this mutation could possibly alter the protein functions [28]. Hence, this study was performed to predict the effect of V727M mutation via a computational approach, along with analyzing altered structural and dynamic insights. For this, *in silico* profiling and molecular dynamic simulation was performed for GNE wt and p.V727M mutant forms to explore the fundamental protein structure as well as the impact of the mutation. We also used DynaMut, a consensus predictor of protein stability based on the vibrational entropy changes predicted by ENCoM and the stability changes predicted by the graph-based signature approach of mCSM. DynaMut analysis also predicted the V727M substitution increases features inducing rigidity of the protein structure. NMA and other methods showed ΔΔG to be in the destabilizing position. The probable functional impacts of these mutations were also gauzed by analysis of the MD trajectories. Simulation results showed that V727M did not seem to induce any significant changes in the structure of the GNE protein. This is also relevant to the findings reported by Chakravorty et al. [22]. In addition, our analysis showed no significant effect of this mutation was seen on post-translational regulation of the GNE protein. Although no structural or post-translational modification was observed due to V727M mutation, the role of such mutation in altering the protein–protein interaction could not be nullified. Interestingly, the leads from our study show that GNE protein could possibly interact with MYC protein (a transcription factor). Such interaction could be altered due to V727M mutation, which could be a major cause in inducing diseased phenotype. Interestingly, MYC is not only a key transcription factor affecting multiple pathways of the cell (growth, proliferation, death, differentiation, metabolism, self-renewal, and pluripotency), but has also been correlated with altered expression of STs [29].

Further, post-translational modification at MYC is well known to control its spatial activity to optimally regulate the expression of various genes in response to extrinsic signals in normal and diseased states. The findings of our study suggest that V727M GNE mutation may alter GNE and MYC protein interaction at the C terminal domain, thus could be leading to altered MYC role in regulating neuromuscular disease-associated gene expression, as well as the expression of ST genes in cells [29]. We hypothesize that the GNE V727M mutation could be altering MYC activity that may decrease the overall STs activity in HIMB disorders. Luo et al., in their studies, have also shown an active involvement of c-MYC in the regulation of multiple miRNAs and lincRNAs that may affect various genes in skeletal muscles via their regulatory effects [24]. Thus the effect of mutations like those of p.V727M could be more impactful in the presence of other altered epigenetic regulators, which could overall drastically affect sialic acid biosynthesis or other pathways leading to HIBM myopathy. Thus, understanding these unexplored miRNAs and lincRNAs associated regulatory networks in HIBM could also further be helpful to understand the mechanism of development of disease.

Overall, the findings from this *in silico* analysis suggest that p.Val727Met mutants although do not confer any significant structural or post-translational changes in the GNE, this mutation seems to impact GNE protein interaction with MYC protein at its C terminal DNA-binding domain. Due to such altered interaction, the c-MYC genome-wide association with muscle cell chromatin is affected, which may affect the overall effect transcription of various genes, miRNAs, and lincRNAs, thereby affecting the expression of STs, neuromuscular disorder-specific genes, overall leading to HIBM myopathy. Thus, enhanced molecular investigation in the future is needed for understanding the role of GNE-associated p.V&27M mutation and its effect on MYC interaction that could help in developing better therapeutic options for effectively targeting HIBM disorder.

## Abbreviations

HIBM: hereditary inclusion body myopathy
GNE-UDP: *N*-acetylglucosamine2-epimerase/*N*-acetylmannosamine kinase
V727M: valine to methionine substitution at 727th amino acid
V696M: valine to methionine substitution at 696th amino acid
V319M: valine to threonine at 319th amino acid
STs: sialyltransferase
PDB: protein Data bank
RMSD: root mean square deviation
RMSF: root mean square fluctuation
ns: nanosecond
PASTAA: predict associated transcription factors from annotated affinities
TFs: transcription factors
FFT: fast Fourier transform
WT: wild type
ps: picoseconds
